# Analgesia for retinopathy of prematurity screening: A systematic review

**DOI:** 10.1111/papr.13138

**Published:** 2022-06-27

**Authors:** Arun J. Thirunavukarasu, Refaat Hassan, Shalom V. Savant, Duncan L. Hamilton

**Affiliations:** ^1^ School of Clinical Medicine University of Cambridge Cambridge UK; ^2^ Corpus Christi College University of Cambridge Cambridge UK; ^3^ Sidney Sussex College University of Cambridge Cambridge UK; ^4^ St John's College University of Cambridge Cambridge UK; ^5^ James Cook University Hospital Middlesbrough UK; ^6^ School of Medicine University of Sunderland Sunderland UK

**Keywords:** analgesic, eye examination, indirect ophthalmoscopy, neonatal, nitrous oxide, opioids, paracetamol, retinopathy of prematurity, screening tools, topical anesthesia

## Abstract

**Background and Aims:**

Premature neonates require regular ophthalmological examination, generally indirect ophthalmoscopy, to screen for retinopathy of prematurity (ROP). Conventional analgesia is provided with topical anesthetic eyedrops and oral sugar solution, but neonates still experience significant pain. Here, the literature base was examined to evaluate the usefulness of other pharmacological analgesics.

**Materials and Methods:**

A systematic review was undertaken, adhering to a PROSPERO preregistered protocol in accordance with PRISMA guidelines (identifier CRD42022302459). Electronic databases were searched for primary research articles on pharmacological pain interventions used for ROP screening in neonates. The primary outcome measure was pain scores recorded using validated pain scoring tools, with and without pharmacological interventions in neonates during eye examination. For analysis, studies were separated into two categories: topical anesthesia and alternative pharmacological treatments.

**Results:**

Eleven studies met the inclusion criteria. Topical analgesia, oral paracetamol, and intranasal fentanyl were found to be effective in reducing the pain of eye examination. Oral morphine and inhaled nitrous oxide had no significant effect on premature infant pain profile (PIPP) scores during indirect ophthalmoscopy.

**Discussion:**

In addition to topical anesthesia, premedication with oral paracetamol is recommended during screening examination for ROP. The routine use of fentanyl is not recommended due to the risk of potential side effects. Non‐pharmacological measures, such as sweet oral solutions and comfort techniques should also be employed. Further research is required to determine whether the use of nitrous oxide has a role, and to develop a safe and effective analgesic strategy to fully ameliorate the pain of ROP screening.

## INTRODUCTION

Retinopathy of prematurity (ROP) is a vasoproliferative disease of the retina characterized by incomplete development of the retinal blood vessels. It is the foremost preventable cause of childhood blindness worldwide.^1^ During normal gestation, development of the retinal vessels proceeds peripherally from the head of the optic nerve, driven by hypoxic conditions in utero. Following premature birth, the associated relative hyperoxia promotes abnormal vascular growth into the vitreous humor, which can lead to fibrovascular retinal detachment.^2^ Early diagnosis and treatment is essential before complications ensue.^1^, ^3^ Treatment options aim to facilitate development of retinal vasculature and prevent pathological intravitreal angiogenesis. These range from destructive cryotherapy or laser photocoagulation of the avascular portion of the retina, to inhibition of angiogenesis with anti‐vascular endothelial growth factor (anti‐VEGF) therapy.^4^ In all cases, prompt management is essential for favorable outcomes.^4^ To identify cases soon enough for effective treatment, regular screening is recommended for infants born before 31 weeks gestational age, or below 1500 g gestational weight.^1^, ^5^, ^6^ Although newer techniques such as digital retinal imaging exist, with potential for artificial intelligence (AI) assistance,^7^ the mainstay of screening is currently indirect ophthalmoscopy to visualize the whole retina.^8^ If present, ROP is classified according to retinal location (zone); degree of disease at the vascular‐avascular junction (stage); and circumferential extent of disease.^9^

Sources of pain during ROP screening include insertion of an eyelid speculum to provide access to the pupil, bright light to illuminate the fundus, and scleral indentation due to manipulation of the eye during examination.^8^, ^10^, ^11^, ^12^ Exposure of neonates to painful procedures may have implications for future health and development,^13^, ^14^, ^15^, ^16^, ^17^ and screening for ROP has been specifically associated with physiological stress and increased rates of apnoeic episodes.^18^ As ROP screening is conducted regularly, until retinal vascularization progresses over a sufficient portion of the retina,^5^ offering safe and effective analgesia for the procedure is important. Efficacy of analgesics in neonates can be evaluated through use of a context‐specific validated scoring tool,^19^ such as the Premature Infant Pain Profile (PIPP), revised in 2014, which uses seven indicators to generate a score out of 21.^20^, ^21^ Scores of 7 or above are an indication for intervention, either comfort measures (7 ≤ PIPP score ≤ 12) or pharmacological analgesia (PIPP score ≥ 13).^19^

Recommendations for pain relief for ROP screening vary, but generally include topical anesthesia, most often proxymetacaine (proparacaine), and nonpharmaceutical measures such as pacifiers, swaddling, and oral sucrose.^6^, ^23^ Many other pharmaceutical and nonpharmaceutical interventions have been described, but no clinically validated intervention or array of interventions has been demonstrated to fully ameliorate the pain of the procedure.^24^ For pain relief in a more general context, some centers recommend paracetamol for PIPP scores greater than 6, and opioids for scores greater than 12,^22^ with topical anesthesia being recommended if feasible.^19^ Specific pharmacokinetic consideration for prescribing analgesics is necessary as screening commences while neonates are still premature in gestational age.^1^, ^5^, ^6^

While previous literature reviews have looked at analgesic strategies for ROP screening, none have looked specifically at pharmacological interventions, and meta‐analysis is complicated by significant heterogeneity in nonpharmacological management and pain assessment.^24^ This review sets out to provide an updated summary of the evidence supporting pharmaceutical analgesic interventions, ranging from well‐established topical anesthesia^25^ to less conventional oral and nasal drugs,^26^, ^27^, ^28^ to evaluate their efficacy without confounding effects of nonpharmacological interventions, which are the mainstay of neonatal pain management, but vary widely between centers. Using PICOS, the objectives of this review are summarized as follows:

**P**articipants: premature neonates undergoing screening for retinopathy of prematurity.
**I**nterventions: pharmacological analgesia used to reduce the procedural pain caused by ROP screening.
**C**omparisons: comparing procedural pain with and without particular pharmacological interventions.
**O**utcomes: procedural pain, assayed with a validated scoring tool such as PIPP.
**S**tudy design: controlled trials, preferably but not necessarily randomized and blinded.

## MATERIALS AND METHODS

**FIGURE 1:**
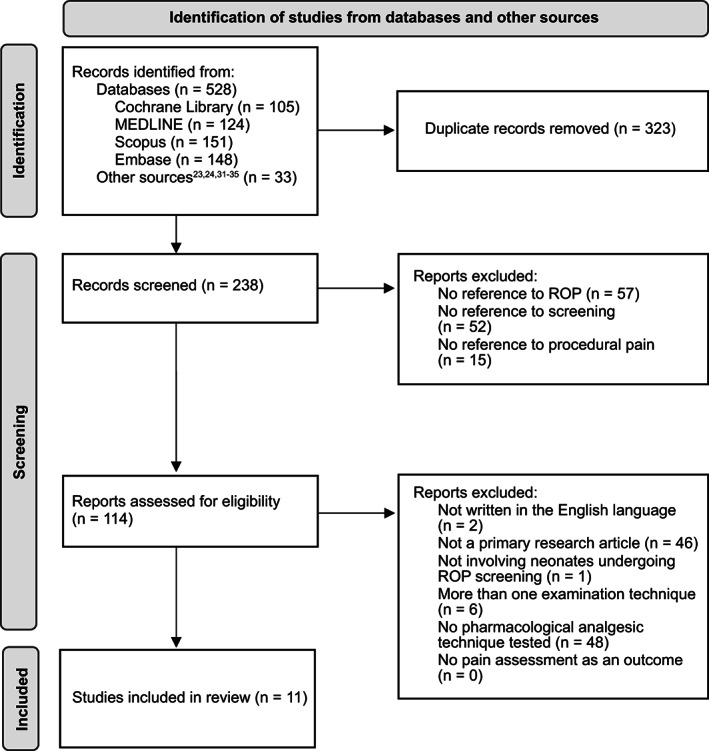
PRISMA flow‐chart depicting how studies were selected for inclusion in this systematic review: Initial search, duplicate exclusion, title and abstract screening, and full‐text screening. Duplicates were removed by a single researcher; screening was conducted by two researchers, with discussion and a third researcher acting as an arbiter to resolve disagreement

The systematic review protocol was prospectively registered in PROSPERO (CRD42022302459),^29^ and PRISMA guidance was adhered to throughout conducting and reporting this review.^30^ A search of The Cochrane Library, MEDLINE (via PubMed), Embase (via OVID), and Scopus was undertaken on January 14, 2022, with no initial restrictions placed on publication date, language, or publication status. The search string was as follows: “retinopathy of prematurity” AND (“analgesia” OR “pain”) in the title, abstract, and/or keywords. Studies were also incorporated from previous reviews on similar topics,^23^, ^24^, ^31^, ^32^, ^33^, ^34^, ^35^ and study protocols from The Cochrane Library were checked for subsequent publications disseminating results. Study selection is illustrated in Figure 1: duplicates were initially removed by a single researcher; title and abstract screening was conducted by two researchers; full text screening was conducted by two researchers. Both researchers appraised every paper at both screening stages; to resolve disagreement, discussion was used to establish consensus, and a third researcher cast a deciding vote if disagreement was still not resolved. Inclusion criteria during title and abstract screening were: (1) Some reference to retinopathy of prematurity (ROP); (2) Some reference to screening or examination; (3) Some reference to procedural pain. The hierarchical criteria for inclusion during full‐text screening were as follows, in descending order:

1. Written in the English language.
2. Is a primary research article.
3. The study population consists of neonates undergoing ROP screening.
4. The same ROP screening technique was used across experimental arms.
5. A pharmacological pain‐relieving intervention was included in the study.
6. Pain assessment was included as an outcome variable.

For articles satisfying the inclusion criteria, two reviewers performed data extraction. Data were extracted solely from text and tables; no extrapolation from graphs was performed. Specifically, the data collected were citation details; the number of subjects, both in total, and in each experimental arm; a comprehensive description of the trialed intervention (ie, drug, route, dose, timing relative to procedure); a comprehensive description of the “base” analgesic interventions and examination technique common to all experimental arms; pain scores during procedure (if multiple pain scores provided, peak mean pain score was extracted) for each experimental arm; and *p* value for *t*‐test or ANOVA comparing the pain scores in each experimental population. A risk of bias analysis was performed by two researchers for each study, with an evaluation as high risk, low risk, or unclear risk in the following seven domains, derived from The Cochrane Collaboration's tool for assessing risk of bias in randomized trials^36^: random sequence generation, allocation concealment, blinding of participants and personnel, blinding of outcome assessment, incomplete outcome data, selective reporting, and other bias (e.g., conflicting interests, but specified if applicable). In cases of disagreement, the third researcher acted as arbiter, following discussion.

Included studies were grouped into two categories based on the intervention being tested: (conventional) topical anesthesia and (less conventional) oral and nasal anesthesia. For each study, examination technique was described comprehensively with an emphasis on analgesic measures (pharmacological and nonpharmacological) undertaken in addition to the trialed method, to illustrate heterogeneity in clinical practice. Due to this confounding heterogeneity, meta‐analysis was excluded; studies exhibit a wide range of baseline interventions, and combine interventions in many different ways.^24^ Instead, a narrative synthesis was organized around summary statistics (outlined above) for each group of studies, and mean changes in PIPP score, weighted by sample size, where multiple studies tested a similar analgesic. Where multiple studies tested a similar intervention, evidence for and against a positive analgesic effect was listed alongside any concerns regarding bias or statistical power. Conclusions were drawn based on the preponderance of evidence, with uncertainty highlighted as encountered. Larger *p* values and smaller effect sizes were interpreted as less certain evidence of a positive analgesic effect, as were studies with a higher risk of bias. To evaluate the risk of publication bias, *p* values were graphed, with a peak at or below *p* = 0.05 being indicative of bias toward “statistically significant” results, perhaps inflating evidence in favor of a positive effect.

## RESULTS

**FIGURE 2:**
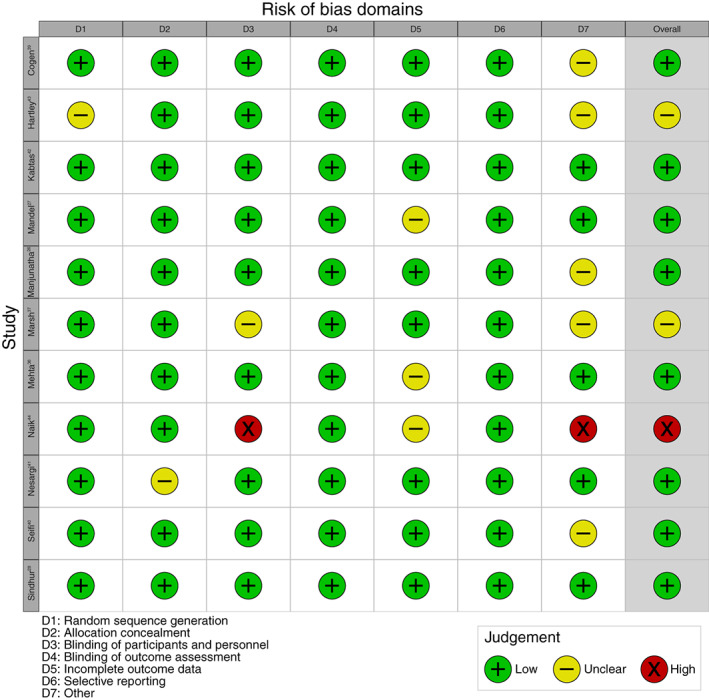
Risk of bias analysis for all of the included studies. Six domains were derived from the Cochrane Collaboration's tool for assessing risk of bias in randomized trials, and studies were also specifically screened for any other potential sources of bias. For each study, two researchers evaluated the risk of bias, with discussion and a third researcher acting as arbiter to resolve any disagreements

The search identified 561 papers, from which 11 studies were selected for further analysis (Figure 1). PICOS characteristics and risk of bias analysis for those 11 studies are presented in Table 1 and Figure 2, respectively. One of the studies was adjudged to exhibit a high risk of bias, due to single‐blind design, lack of placebo control, and loss of data due to poor quality videos.^44^ Two studies were categorized as unclear in terms of risk of bias, one due to use of a minimization sequence rather than randomization and early trial cessation, and another due to potential early unblinding and early trial cessation.^37^, ^43^ For further analysis, studies were grouped according to whether they trialed topical anesthesia instilled into the conjunctival sac, or analgesics via the oral or nasal routes.

**TABLE 1:** PICOS table summarizing the 11 studies included in the systematic review. Semi‐colons separate distinct experimental arms. Procedures are described in as much detail as provided by the study full‐text

Citation	Participants	Interventions	Comparisons	Outcomes	Study design
Marsh et al, 2005^27^	22 premature neonates undergoing indirect ophthalmoscopy with scleral depression and wire speculum. All patients swaddled for several minutes before examination and held by a nurse during examination.	Proparacaine HCl 0.5% (2 drops immediately prior to examination).	NaCl 0.5% (2 drops immediately prior to examination).	PIPP measured before (5 min, 1 min) examination and during speculum placement.	Randomized double blind placebo‐controlled crossover; two arms.
Manjunatha et al, 2009^26^	18 premature neonates. All patients given one drop 0.5% proparacaine 0.5% in each eye, 5 min before examination.	Morphine sulfate 200 μg kg^−1^ (oral dose 60 min before examination); paracetamol 30 mg kg^−1^ (oral dose 60 min before examination).	Placebo 2 ml kg^−1^ (oral dose 60 min before examination).	PIPP measured before (5 min) and after (5 min, 30 min, 60 min, 120 min, 180 min).	Randomized double blind placebo‐controlled crossover; three arms.
Mehta et al, 2010^38^	40 premature neonates undergoing indirect ophthalmoscopy with lid speculum and scleral depression. All patients given nonnutritive pacifier and swaddled during examination.	Proparacaine HCl 0.5% (drops during examination).	Saline (drops during examination).	PIPP measured before (1 min) and after (1 min, 5 min) examinations commenced.	Randomized double blind placebo‐controlled crossover; two arms.
Cogen et al, 2011^39^	34 premature neonates undergoing indirect ophthalmoscopy with scleral depression.	Proparacaine HCl 0.5% (drops during examination)	Artificial tears (drops during examination).	PIPP measured after speculum insertion, during initial visualization of the retina, and after scleral depression.	Randomized double blind placebo‐controlled crossover; two arms.
Mandel et al, 2012^27^	40 premature neonates undergoing indirect ophthalmoscopy. All infants swaddled by a nurse throughout examination; one drop proparacaine 0.5% in each eye 1 min before examination; 24% sucrose administered orally at the nurse's discretion, starting 1 min before local anesthetic.	50% oxygen and 50% nitrous oxide gas mixture (nasal cannula initiated 5 min before examination).	EMONO 50% oxygen 50% nitrogen gas mixture (nasal cannula initiated 5 min before examination).	PIPP measured after speculum insertion and 30 min after examination.	Randomized double blind placebo‐controlled crossover; two arms.
Seifi et al, 2013^40^	120 premature neonates undergoing ROP screening. All infants given tetracaine 1% eyedrops prior to examination of each eye.	Paracetamol 15 mg kg^−1^ (oral dose 30 min before examination) and sterile water 0.2 mL (orally administered during examination).	25% sucrose 0.2 ml (orally administered during examination); sterile water 0.2 ml (orally administered during examination).	PIPP measured during the first and last 45 s of each examination.	Randomized double blind placebo‐controlled crossover; three arms.
Nesargi et al, 2015^41^	20 premature neonates undergoing indirect ophthalmoscopy. All infants given proparacaine 0.5% drops 10 min prior to examination of each eye.	Proparacaine HCl 0.5% (1 eye‐drop immediately prior to examination)	25% dextrose 2 mL (oral dose administered 10 min before examination).	PIPP measured during examination of the left eye.	Randomized double blind crossover; two arms.
Kabataş et al, 2016^42^	114 premature neonates undergoing ROP screening. All infants given 0.5% proparacaine applied 30s before examination.	Paracetamol 15 mg kg^−1^ (single oral dose 60 min before examination)	15 ml kg^−1^ sterile water (single oral dose 60 min before examination).	PIPP measured during examination of the first eye.	Randomized double blind placebo‐controlled crossover; two arms.
Hartley et al, 2018^43^	31 premature neonates undergoing indirect ophthalmoscopy with scleral indenter and eyelid speculum. All infants swaddled before procedure and given 0.5% proxymetacaine drops before insertion of eyelid speculum.	Morphine sulfate 100 μg kg^−1^ (single oral dose 60 min before examination).	Placebo 100 μg kg^−1^ (administered via oral syringe or nasogastric tube 60 min before examination).	PIPP‐R measured 30s after speculum removed post‐examination.	Randomized double blind placebo‐controlled crossover; two arms.
Sindhur et al, 2020^28^	111 premature neonates undergoing indirect ophthalmoscopy with scleral indenter and eyelid speculum. All infants given 0.5 mL of oral sucrose 24% 1 min prior to examination and 0.5% proparacaine 30s prior.	Fentanyl 2 μg kg^−1^ (intranasal administration 5 min before examination).	Saline 0.3 ml (intranasal administration 5 min before examination).	PIPP‐R measured during and after (1 min, 5 min) examination.	Randomized double blind placebo‐controlled crossover; two arms.
Naik et al, 2021^44^	120 premature neonates undergoing indirect ophthalmoscopy with scleral indenter and eyelid speculum. All infants given proparacaine drops prior to examination and swaddled during procedure.	Paracetamol 15 mg kg^−1^ (single oral dose 30 min before examination)	Conventional analgesia only; expressed breast milk 2 mL (orally administered 2 min before examination).	PIPP measured before (20s), during, and after (2 min) examination.	Randomized single blind crossover; three arms.

Abbreviations: PIPP, premature infant pain profile; PIPP‐R, premature infant pain profile revised.

### Topical anesthesia

Three of four studies evaluating topical anesthesia, proxymetacaine 0.5% in every case, reported lower pain scores in infants treated with topical anesthesia compared with placebo, with weighted mean PIPP reduction of 1.66, and *p* values ranging from 0.001 to 0.1 (Table 2).^27^, ^38^, ^39^, ^41^ One study compared topical anesthesia with 0.5% proxymetacaine to sweet oral solution and found no statistically significant difference (*p* = 0.165) between them in terms of pain relief.^41^ In all cases, pain relief was incomplete, with mean PIPP scores greater than 7 in every experimental arm; one study even exhibited mean PIPP scores greater than 14, beyond a recognized threshold for additional pharmacological intervention. In one study, mean PIPP scores were calculated from more granular data that provided average scores for separate eyes in both experimental groups.^41^

**TABLE 2:** Results of randomized trials evaluating topical anesthesia for ameliorating the pain of ROP screening

Citation	*N*	Experimental arms	Pain scores	*p*
Cogen et al, 2011^39^	34	(A) Proxymetacaine	PIPPA = 10.4	0.1
		(B) Artificial tears	PIPPB = 12.0	
Marsh et al, 2005^27^	22	(A) Proxymetacaine	PIPPA = 11	0.001
		(B) Saline drops	PIPPB = 13.5	
Mehta et al, 2010^38^	40	(A) Proxymetacaine	PIPPA = 10.375	0.027
		(B) Saline drops	PIPPB = 11.725	
Nesargi et al, 2015^41^	20	(A) Proxymetacaine	PIPPA = 14.75	0.165
		(B) Sweet taste	PIPPB = 14.55	

Abbreviation: PIPP, premature infant pain profile.

### Alternative pharmacological treatments

Various alternative analgesics have been trialed: paracetamol (acetaminophen), morphine, fentanyl, and nitrous oxide (Table 3).^26^, ^27^, ^28^, ^40^, ^42^, ^43^, ^44^ Administration routes included oral, intranasal, and inhaled gas, with no intravenous medication trialed. Evidence for paracetamol is generally positive, although no statistically significant effect was found in a trial comparing 15 mg kg^−1^ paracetamol given orally 30 min preprocedure to both a breastmilk prefeed and no‐feed control, in a trial deemed to exhibit a high risk of bias.^44^ In contrast, positive effects were recorded for 15 mg kg^−1^ paracetamol given 60 min preprocedure versus water control,^42^ and 15 mg kg^−1^ paracetamol given 30 minutes preprocedure versus water and oral sucrose.^40^ Another study comparing 20 mg kg^−1^ paracetamol 60 minutes preprocedure to placebo exhibited a limited positive effect but did not reach the authors' threshold for statistical significance, perhaps due to a much smaller sample size, and assessment of pain after the procedure, rather than during examination.^26^ In these studies, paracetamol conferred a weighted mean PIPP reduction of 2.18, *p* values ranging from 0.001–0.75.

**TABLE 3:** Results of randomized trials evaluating alternative pharmaceuticals, defined as anything other than topical anesthesia, for ameliorating the pain of ROP screening

Citation	*N*	Experimental arms	Pain scores	*p*
Kabataş et al, 2016^42^	114	(A) TA and paracetamol	PIPP_A_ = 12	0.01
		(B) TA and water	PIPP_B_ = 14	
Naik et al, 2021^44^	120	(A) TA and paracetamol	PIPP_A_ = 15.83	0.72
		(B) TA and breastmilk/formula prefeed	PIPP_B_ = 15.44	
		(C) TA	PIPP_C_ = 15.74	
Seifi et al, 2013^40^	120	(A) TA and sweet taste	PIPP_A_ = 12.9	<0.001
		(B) TA and paracetamol	PIPP_B_ = 9.0	
		(C) TA and water	PIPP_C_ = 13.7	
Manjunatha et al, 2009^26^	18	(A) TA and paracetamol	PIPP_A_ = 4.600	0.083
		(B) TA and morphine	PIPP_B_ = 3.500	
		(C) TA and placebo	PIPP_C_ = 6.167	
Hartley et al, 2018^43^	31	(A) TA and morphine	PIPP_A_ = 11.1	0.66
		(B) TA and placebo	PIPP_B_ = 10.5	
Sindhur et al, 2020^28^	111	(A) TA and sucrose and fentanyl	PIPP_A_ = 8.3	<0.001
		(B) TA and sucrose and saline	PIPP_B_ = 11.5	
Mandel et al, 2012^27^	40	(A) TA and sweet taste and N_2_O/ O_2_ gas	PIPP_A_ = 8.5	0.94
		(B) TA and sweet taste and N_2_/O_2_ gas	PIPP_B_ = 8.4	

Abbreviations: N_2_, nitrogen; N_2_O, nitrous oxide; O_2_, oxygen; PIPP, premature infant pain profile; TA, topical anesthesia.

Three studies tested the effect of opioid analgesics. One, using 200 μg kg^−1^ morphine sulfate, given orally 1 h preprocedure, exhibited a positive effect but failed to meet the authors' criteria for statistical significance, likely due to a small sample size of 18.^26^ A larger study of 31 infants, which administered 100 μg kg^−1^ morphine sulfate given orally 1 h preprocedure, found no significant difference between morphine and placebo groups.^43^ A weighted mean PIPP reduction of 0.60, with *p* values of 0.083 and 0.66 do not provide convincing evidence for the efficacy of oral morphine. However, in the single study testing intra‐procedural 2 μg kg^−1^ intranasal fentanyl, a significant and relatively large positive effect was noted.^28^

Finally, one study tested the use of an oxygen and nitrous oxide gas mixture delivered via a nasal cannula during the procedure in a cohort of 40 neonates, finding almost identical pain levels between this group and a control group treated with a placebo consisting of an oxygen and nitrogen gas mixture.^27^

As with the topical anesthesia trials, no intervention conferred complete pain relief, as the mean PIPP scores were greater than 7 in every experimental arm. While most evidence points toward paracetamol and nasal fentanyl having a significant analgesic effect, the evidence suggests oral morphine and nitrous oxide gas have little to no effect.

## DISCUSSION

This review reveals that a range of pharmacological analgesics have been trialed as measures to reduce the pain associated with ROP screening. There is more evidence for topical anesthesia and paracetamol, with fewer studies exploring the use of opioids or nitrous oxide. The preponderance of evidence supports positive analgesia being conferred by intra‐procedure topical proxymetacaine, preprocedure oral paracetamol, and intra‐procedure intranasal fentanyl, whereas all published evidence suggests that preprocedure oral morphine and intra‐procedure inhaled nitrous oxide do not provide effective pain relief. The evidence was generally concordant with the above conclusions, although one of four studies testing proxymetacaine did not exhibit a significant effect, and two of four studies testing paracetamol similarly found no significant effect. In the single study testing nitrous oxide, it is difficult to determine the actual inspired nitrous oxide fraction using this delivery method. While it is possible to deliver Entonox® more effectively using an anesthetic breathing circuit, this can be challenging during indirect ophthalmoscopy.^27^

Risk–benefit analysis is necessary to determine which apparently effective analgesics are suitable for routine use in ROP screening. Of the three effective agents described above, topical anesthesia is already widely utilized, and is mentioned in national guidelines.^5^ Preprocedure paracetamol is not so widely used, but is already indicated for lower pain levels than local anesthesia,^19^ and is generally considered safe. Recommended doses of 20–25 mg kg^−1^ paracetamol are higher than three of four studies reviewed here, and carry very low risks of hepatic or renal toxicity, though lower doses may be appropriate to account for preterm infants with lower clearance.^45^ Fentanyl is a potent opioid reserved for more severe pain. It is generally used to induce deep sedation or anesthesia,^19^ and its side effects include respiratory depression, bradycardia, and chest wall rigidity.^46^, ^47^ Use of fentanyl is generally restricted to specialists in anesthesia, and it may not be a justifiable choice for routine use, despite exhibiting effectiveness in a single study.^28^ To further reduce pain, clinicians could instead focus on nonpharmacological interventions, such as swaddling, nesting, and oral sugar solution (eg, sucrose, dextrose).^24^, ^31^, ^32^, ^34^, ^48^

It is justifiable to generalize conclusions made here to all neonatal eye examinations, although ROP screening is one of the most common reasons they are undertaken.^49^ However, conclusions cannot be extended to ophthalmological procedures, including cryotherapy and laser treatment (which may be indicated in ROP). This review is limited by the lack of quantitative meta‐analysis, not undertaken due to difficulties in combining studies with different examination techniques and analgesic measures outside the tested intervention, and relatively small number of studies, making network meta‐analysis overly reliant on modeled results. Weighted mean PIPP reductions calculated above may not accurately represent the effect of a given analgesic for similar reasons. The review is also limited by the relatively small number of studies exploring the effects of pharmacological analgesics, of which most had sample sizes lower than 50. The distribution of studies across the full possible range of *p* values, despite a peak below *p* = 0.05, suggests that publication bias is minimal. However, there are relatively few studies testing any of the above interventions, and sample sizes frequently small enough to raise concerns of a lack of statistical power, and increased risk of random significant results.

In summary, our recommendation based on the above evidence would be to incorporate preprocedure oral paracetamol and intra‐procedure topical anesthesia with proxymetacaine to ameliorate the pain of ROP screening. These interventions should be combined with nonpharmacological measures such as swaddling, nesting, and oral sugar solution, which have proven efficacy and form the basis of pain management in this setting.^24^ Further investigation is necessary to engineer analgesic solutions, either pharmacological or otherwise, avoiding the side effects and sedation associated with opioids.^24^ Other unanswered questions include the optimal dosage of paracetamol and whether the use of nitrous oxide is effective. Trials should focus on robust design to avoid bias and maximize reliability, as well as a prospective power analysis to ensure a sufficient sample size is tested.

## AUTHOR CONTRIBUTIONS

AJT conceived, designed, and led coordination on the study. AJT undertook preliminary literature searches and designed the search strategy. AJT, RH, and SS contributed to screening and study selection. AJT, RH, and SS conducted data extraction and risk of bias analysis. AJT and RH performed data analysis and produced figures. AJT and DLH discussed the implications of results and prepared the manuscript. DLH provided advice throughout the project and edited the final draft. All authors approved the final draft manuscript submitted for publication.

## CONFLICT OF INTEREST

The authors declare they have no conflicts of interest to disclose.

## Data Availability

All data presented in the manuscript can be provided upon request.
